# Copper homeostasis and cuproptosis in central nervous system diseases

**DOI:** 10.1038/s41419-024-07206-3

**Published:** 2024-11-21

**Authors:** Zhipeng Zhu, Min Song, Jianxun Ren, Lirong Liang, Guohua Mao, Min Chen

**Affiliations:** 1 Department of Neurosurgery, The Second Affiliated Hospital, Jiangxi Medical College, Nanchang University, Nanchang, Jiangxi Province China; 2 Department of Neurosurgery, Shangrao People’s Hospital, Shangrao, China

**Keywords:** Cell death, Cell death in the nervous system

## Abstract

Copper (Cu), an indispensable micronutrient for the sustenance of living organisms, contributes significantly to a vast array of fundamental metabolic processes. The human body maintains a relatively low concentration of copper, which is mostly found in the bones, liver, and brain. Despite its low concentration, Cu plays a crucial role as an indispensable element in the progression and pathogenesis of central nervous system (CNS) diseases. Extensive studies have been conducted in recent years on copper homeostasis and copper-induced cell death in CNS disorders, including glioma, Alzheimer’s disease, Amyotrophic lateral sclerosis, Huntington’s disease, and stroke. Cuproptosis, a novel copper-induced cell death pathway distinct from apoptosis, necrosis, pyroptosis, and ferroptosis, has been identified as potentially intricately linked to the pathogenic mechanisms underlying various CNS diseases. Therefore, a systematic review of copper homeostasis and cuproptosis and their relationship with CNS disorders could deepen our understanding of the pathogenesis of these diseases. In addition, it may provide new insights and strategies for the treatment of CNS disorders.

## Facts

- Copper regulates a wide range of physiological processes as an essential micronutrient.
- Copper-induced cell death (Cuproptosis) is thought to be involved in the pathogenesis of central nervous system diseases.
- In recent years, therapies for central nervous system diseases targeting cuproptosis have been developed but have limitations.

## Questions

- Is programmed cell death involved in copper-induced cell death?
- How loss of Fe-S clusters protein is involved in inducing cascading cell damage?
- How to determine the optimal copper concentration for the treatment of different CNS disorders?

## Introduction

As an essential micronutrient for the survival of living organisms, copper (Cu) is widely involved in a variety of fundamental metabolic activities, including mitochondrial respiration, oxidative stress, inflammatory response, and synthesis of various biomolecules [1]. The concentration of Cu in the human body is maintained at a relatively low level, typically ranging from 50 to 120 mg, and is primarily distributed in the brain, liver, and bones [2]. Despite its low concentration, Cu plays a crucial role as an indispensable element in the progression and pathogenesis of central nervous system (CNS) disorders [3, 4]. In recent years, there has been extensive research on copper metabolism and copper-induced cell death in CNS disorders such as glioma, Alzheimer’s disease (AD), Amyotrophic lateral sclerosis (ALS), Huntington’s disease (HD), and stroke [5–7].

**Fig. 1:**
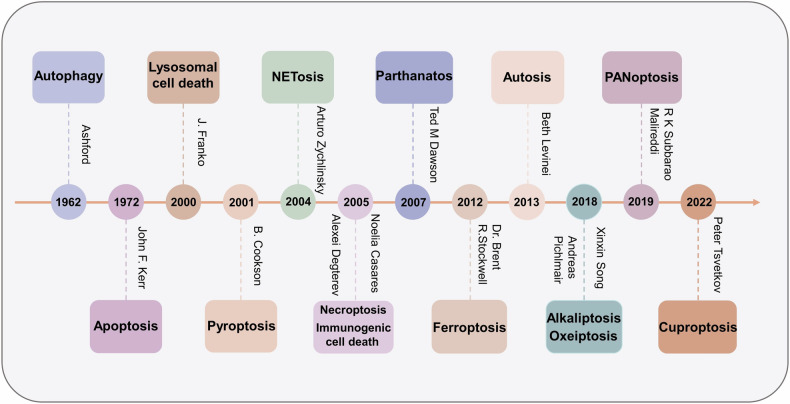
Timeline of the history and milestones of cell death modalities. The review summarizes the time of the discovery of 12 methods of cell death, including cuproptosis, and associated important figures.

Cu serves as a crucial catalytic cofactor for various physiological processes, and the proper development of the nervous system is heavily reliant on its presence [8]. Cuproptosis, which has been recognized as a novel cell death pathway dependent on copper [9], may be tightly linked to the pathogenic process of several CNS disorders [10–12]. Intracellular copper concentration is tightly regulated at a low level, as excessive amounts of Cu can lead to cytotoxicity and even cell death [13]. Consequently, the absorption, distribution, and elimination of copper are strictly controlled to maintain homeostasis. For a long time, the exact mechanism that copper-induced cell death was unclear, until last year when a study demonstrated that cuproptosis is a new cell death pathway, distinct from apoptosis, necrosis, pyroptosis, and ferroptosis [14]. We also provide a summary of the many modes of cell death, including cuproptosis, whose discovery has fueled the field (Fig. 1). In this review, we summarized the current literature focused on copper homeostasis and cuproptosis in CNS disease. In addition, we also discuss potential therapeutic strategies that target cuproptosis, which provides further insight and ideas for the clinical approach to the treatment of cuproptosis-related CNS disorders.

## Systemic copper homeostasis

As an important transition metal, Cu is present in nearly all living organisms. Extensive research has evinced that copper often acts as a cofactor for various metabolic enzymes and participates in the regulation of several physiological processes [15]. However, due to the metal toxicity of Cu, the systemic Cu levels are maintained within a fairly restricted range to support normal biochemical processes. The recommended daily intake of copper for adults is 0.8–2.4 mg, primarily obtained from foods high in Cu, such as animal organ meats and shellfish, to maintain systemic copper homeostasis [16]. In the mammalian digestive tract, absorption of dietary copper is majorly dependent on the small intestine and duodenum, mediated through Cu transport protein 1 (CTR1) or called solute carrier family 31 member (SLC31A1) located on the apical side of intestinal epithelial cells [17]. Scholars commonly agree that CTR1-mediated Cu uptake is the major route of Cu acquirement by peripheral tissues or organs [18]. In addition, CTR1 expression was found to be regulated by Cu, which was down-regulated in the presence of Cu excess and increased in the presence of Cu deficiency, indicating the existence of a negative feedback loop with CTR1 regulation [19]. Extracellular copper ions usually exist in the small intestine as divalent copper ions; however, divalent copper ions cannot be directly absorbed by intestinal epithelial cells [20]. Under the facilitation of activated metalloreductases six-transmembrane epithelial antigen of the prostate (STEAP) and duodenal cytochrome b (DCYTB), divalent Cu is reduced to monovalent Cu, which is the ionic state of Cu transported by CTR1 [21]. Copper is transported to another side of the intestinal epithelial cells by antioxidant 1 copper chaperone (ATOX1) and then to the blood via adenosine triphosphatase copper transporting alpha (ATP7A) [22]. Copper ions in the blood participate in the circulation mainly in the bound form with proteins rather than the free form. Among them, about 75% of copper ions are bound to ceruloplasmin (CP) in a non-exchangeable manner, and the rest of the exchangeable copper is equilibrated between human serum albumin (25%) and histidine (0.2%) [23]. Copper entering the portal circulation bound to these plasma proteins is transported to organs and tissues.

The liver is the main organ of copper storage in the body and also the major organ of copper excretion in the body. CTR1 mediates Cu uptake in hepatocytes, in addition, Cu can be delivered to specific proteins via intracytoplasmic Cu chaperones or chelated for storage with cytosolic metallothioneins (MT1, MT2) [24]. These Cu Chaperones are primarily COX17 (Cu chaperone for cytochrome c oxygenase, which delivers Cu to CCO), CCS (Cu chaperone for superoxide dismutase, delivers Cu to SOD1), and ATOX1 (delivers Cu to ATP7A and ATP7B). Excess copper can be excreted through the ATOX1/ATP7B/ceruloplasmin pathway by secreting into the bile as vesicles, which is an important mode of endogenous Cu excretion [24]. Moreover, little Cu is excreted in the feces or the form of unabsorbed metal ions, and the amount of Cu lost in sweat, urine, and menstruation is minimal. When the level of Cu in peripheral tissues and organs is inadequate to maintain normal physiological function, ATP7B located in the Golgi apparatus transports Cu which is stored in hepatocytes to the bloodstream and bound to plasma proteins to enter the systemic circulation [22]. Cu entering the somatic circulation is transported to specific tissues and organs, where it exerts its catalytic effects in a variety of physiological processes, including mitochondrial energy production, redox homeostasis, neuro repair, and extracellular matrix remodeling [25].

In summary, regulation of systemic copper homeostasis is primarily dependent on duodenal absorption and biliary excretion. With high Cu intake, CTR1-mediated absorption from the gastrointestinal tract is reduced and Cu excretion is increased. Excessive copper intake induces degradation and endocytosis of CTR1/SLC31A1 protein, and SP1 (Sp1 transcription factor) is involved in regulating this process [26, 27]. In contrast, when Cu intake is inadequate, endogenous Cu excretion through the bile decreases and retained Cu increases.

## Copper homeostasis in cells

Copper ions that enter the cell are transported by copper chaperones to different cellular subcompartments, including the cytoplasm, Golgi, mitochondria, and nucleus, where they participate in the regulation of a multitude of intracellular physiological processes. Based on the kinetics of intracellular copper, it can be divided into two main categories, the stationary copper pool and the unstable copper pool [28]. Compared to the stationary pool, copper in the unstable pool has better bioavailability. The maintenance of copper homeostasis at the cellular level is strictly regulated by a network of copper-dependent proteins, consisting of cuproenzymes, copper chaperones, and membrane transporter proteins, which regulate Cu import, export, and intracellular utilization [29].

### Copper uptake into cells

Divalent copper ions arriving at the cell surface are reduced to monovalent copper icons catalyzed by STEAP proteins, two His-Met clusters in the extracellular amino terminus of CTR1 bind and maintain the reduced state of the copper, which is subsequently transported into the cell [30]. Entering the cell, Cu is delivered to different protein targets with the assistance of different protein transporters. Most of the Cu cellular uptake is dependent on CTR1, and the expression of CTR1 is regulated in a Copper-dependent mode. CTR1 expression was down-regulated under Cu overload and increased under Cu-deficient conditions, which indicates the existence of a negative feedback mechanism for CTR1 to maintain normal Cu homeostasis [31]. A previous study demonstrated that mice with CTR1 gene deficiency or knockout have significantly increased perinatal mortality [32]. Copper homeostasis imbalance results in oxidative stress which damages almost all cellular structures, and as a high-affinity copper transporter protein, CTR1 plays a crucial role in maintaining copper homeostasis.

### Intracellular Cu utilization

In the cytoplasm, the copper chaperone CCS delivers copper to superoxide dismutase 1 (SOD1). SOD1 is an important antioxidant protein, which is mainly distributed in the cytoplasm and has a small portion located in the mitochondrial intermembrane space (IMS) [33]. With the involvement of oxygen, CCS can promote the formation of SOD1 disulfide bonds, which is crucial for the maintenance of spatial conformation and the activity of biological enzymes [34]. Through this regulatory mechanism, SOD1 can detoxify the superoxide produced by the electron transport chain (ETC) in IMS, maintain the stability of reactive oxygen species (ROS), and avoid oxidative stress injury caused by copper overload in vivo [35]. Similar to CTR1, there is also a negative feedback mechanism for CCS expression in the maintenance of copper homeostasis, with increased inactivation of CCS proteins when intracellular Cu levels are excessive, and increased CCS expression when intracellular Cu levels are low.

As a copper chaperone, ATOX1 binds Cu via two cysteine residues and carries it to the trans-Golgi network (TGN) [36]. Cu transported to the TGN binds to ATP7A/B and promotes the synthesis of cuproenzymes such as ceruloplasmin, lysyl oxidase, and tyrosinase [37]. The expression of ATP7A/B is thought to be tissue-specific, with ATP7A being expressed in most organs and tissues except the liver, whereas ATP7B is predominantly found in the liver [38]. In addition, ATP7A/B is also closely related to intracellular copper export, to avoid copper toxicity, excess intracellular copper binds to the metal-binding sites of ATP7A/B and is exported to the extracellular compartment with the involvement of ATP [39]. As natural intracellular chelators of copper ions, MT and glutathione can bind to copper to prevent cytotoxicity induced by excess copper.

**Fig. 2:**
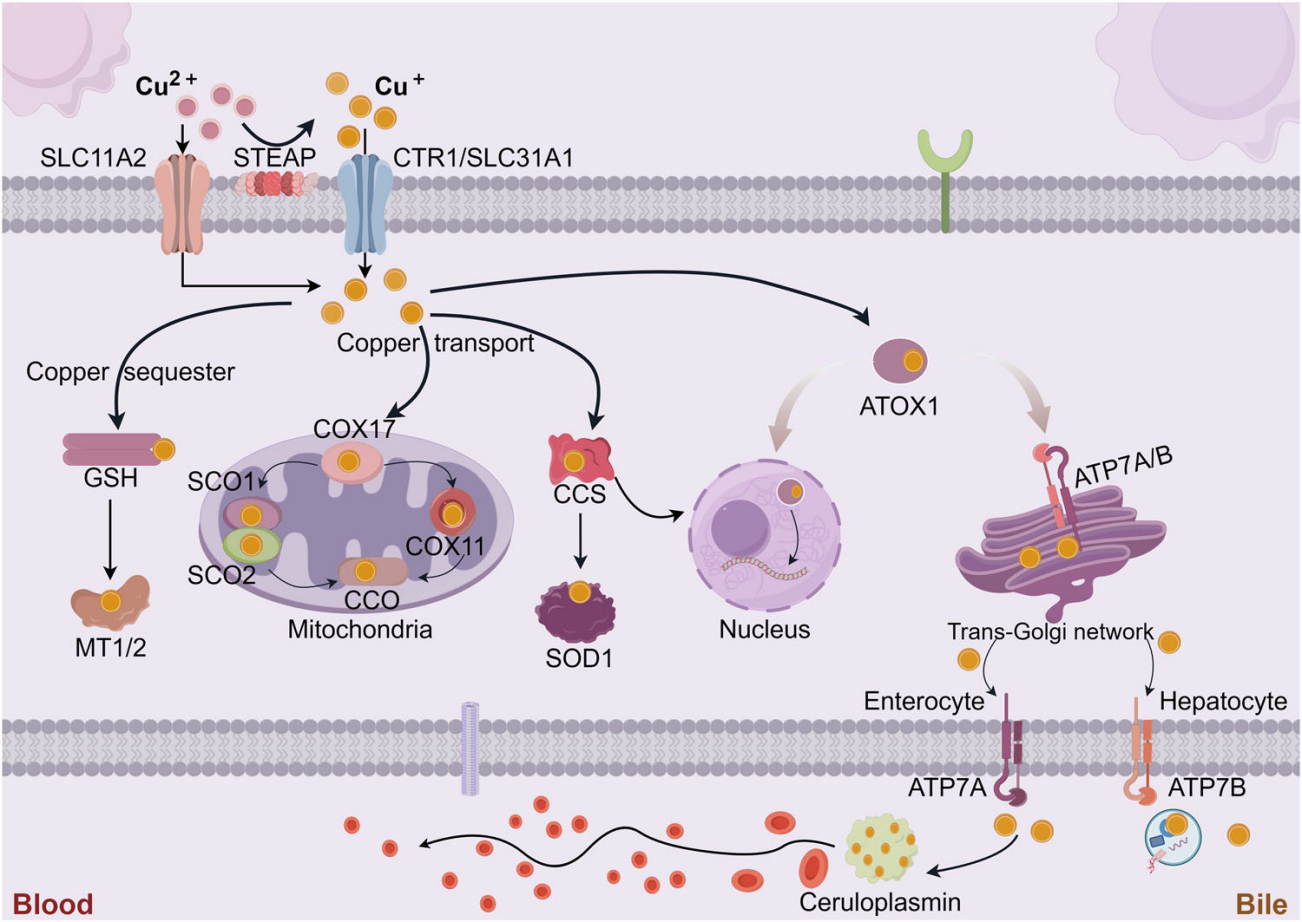
Mammalian copper metabolism at the molecular level. Cu^2+^ is reduced to Cu^+^ with the participation of STEAP, and CTR1 is highly specific for Cu^+^ uptake. Copper-transporting ATPases are located in the TGN, where they pump Cu^+^ from the cytoplasm into the TGN lumen. These copper-transporting ATPases fuse with the plasma membrane to export Cu^+^ when intracellular Cu^+^ increases. Cu^+^ can be sequestered by MT1/2 for storage. Copper is transported by ATP7A through the basolateral membrane of enterocytes into the portal circulation, where it reaches the liver, the primary organ for storing copper. Via ATP7B, extra copper in liver cells is released as vesicles into bile. Cu^+^ is transported by CP to the whole-body system. In addition, Cu^+^ is carried to the nucleus through ATOX1, where it binds to transcription factors to promote the expression of certain genes. To trigger the function of the respiratory chain’s enzymes, COX17 carries Cu^+^ to the SCO1, SCO2, and COX11 which carry copper, and then delivers it to CCO. Cu^+^ can be transferred from CCS to SOD1.

Other than binding to cytoplasmic-specific proteins, copper can also target mitochondria and bind to relevant copper chaperone proteins engaged in various processes such as mitochondrial respiration, protein synthesis, and secretion [40]. Cytochrome C oxidase copper chaperone 17 (COX17), which is located in the IMS, transports Cu from the cytoplasm to the IMS and further delivers Cu to the cysteine residues of the secondary copper-carrying protein synthesis of cytochrome C oxidase 1/2 (SCO1, SCO2) and forms a disulfide bond [41]. Through both the SCO1 or cytochrome C oxidase copper chaperone (COX11) pathways, copper is delivered to the cytochrome C oxidase (CCO) to activate the activity of enzymes in the mitochondrial respiratory chain [42] (Fig. 2). Copper homeostasis is maintained with the involvement of the above copper metabolism-related proteins (Table 1).

**Table 1:** Copper metabolism-related regulatory proteins.

Protein	Type	Function	Ref
CTR1	Cu transport protein 1	Transmembrane transport of copper ion	[14]
STEAP	Metalloreductase	Reduction of divalent copper ions to monovalent copper ions	[143]
DCYTB	Metalloreductase	Promote the conversion of copper ions to the reduced state	[144]
ATOX1	Antioxidant 1 copper chaperone	Transports cytoplasmic copper to organelles	[145]
ATP7A/B	Copper‐transporting ATPases	Regulation of copper ion transport	[146]
CP	Cuproenzymes	Regulates copper distribution	[15]
MT1/2	Metallothionein	Chelated copper reduces it’s metal toxicity	[17]
GSH	Copper chelator	Chelated copper reduces it’s metal toxicity	[23]
COX17	Copper chaperone	Regulates mitochondrial copper	[22]
CCO	Cuproenzymes	Regulates cellular biochemical reactions	[31]
CCS	Copper chaperone	Regulates transport of copper	[34]
SOD1	Cuproenzymes	Antioxidant damage	[145]

*STEAP* six-transmembrane epithelial antigen of the prostate, *DCYTB* duodenal cytochrome b, *CP* ceruloplasmin, *GSH* glutathione, *COX17* cytochrome c oxidase copper chaperone, *CCO* cytochrome C oxidase, *CCS* Cu chaperone for superoxide dismutase, *SOD1* superoxide dismutase 1.

### Importance of copper homeostasis at the cellular level

Overall, copper homeostasis plays an important role at the cellular level. Extracellular Cu^2+^ participates in the regulation of the physiological functions of various cytokines, Cu^2+^ that arrives at the cell membrane is reduced to Cu^+^, which alters the structure of membrane proteins to change their active state. Cytoplasmic Cu^+^ contributes to the maintenance of normal physiological functions of various organelles and avoids cytotoxicity produced by peroxidation [43]. The binding of Cu^+^ to transcription factors in the nucleus can regulate gene expression and protein synthesis [44]. The maintenance of intracellular copper homeostasis is mainly dependent on the above copper chaperone proteins and copper transporter proteins, the imbalance of copper homeostasis will lead to cellular metabolic disorganization and even death [45].

## Copper induced cell death

There have been a lot of studies on copper, and in the 1970s, researchers found high levels of copper-induced fibroblast cell death [46]. However, none of these studies revealed the underlying mechanism of copper-induced cell death. Copper-induced cell death has always been controversial, most researchers attribute cell death to programmed cell death (PCD) such as apoptosis, necrosis, and autophagy, and experimental results provide some basis for this opinion. It wasn’t until 2022 that Tsvetkov with his colleagues [14] clarified the exact mechanism of copper-induced cell death, which they called “cuproptosis”, and a whole new era of research on copper-induced cell death was launched.

### Copper ionophores induce a new form of regulated cell death

The study found that cuproptosis is a completely new form of cell death that is distinct from known apoptosis, necrosis, pyroptosis, and ferroptosis [9]. Sufficient evidence indicated that intracellular copper accumulation was the primary trigger for copper death, and classical cell death markers such as caspases were not detected when Cu ion carrier induced cell death [47]. At first, the authors investigated whether the toxicity of copper ionophores (elesclomol, disulfiram (DSF), and NSC319726), especially elesclomol (ES), is mediated by the currently known modalities of cell death. Pharmacological inhibition of some previously known regulatory cell deaths, including apoptosis (knockdown of BAK1 and BAX genes or using caspase inhibitor ZVAD-FMK), necroptosis (Necrostatin-1), pyroptosis (Necrosulfonamide), oxidative stress (N-Acetylcysteine), and ferroptosis (Ferrostain-1 and Deferasirox), found that Cu carrier induced cell death hardly intervened, suggesting the uniqueness of cuproptosis. As for why the cell death induced by copper ionophores did not follow the known PCD pathways such as apoptosis, it is not clear, which leaves thoughts for follow-up research.

### Importance of mitochondrial respiration in copper-induced cell death

Tsvetkov et al. [14] also observed that the mode of cell death induced by Cu ionophores was highly dependent on mitochondrial respiration, and cells dependent on mitochondrial respiration are nearly 1000-fold more sensitive to copper ion carriers than cells undergoing glycolysis. It follows that mitochondria are the primary target of cuproptosis, and when the mitochondrial membrane is damaged by oxidative stress, the function of enzymes in the tricarboxylic acid (TCA) cycle is impaired. Reduced CCO activity and inhibition of the TCA cycle were found to lead to the inactivation of aconitase in Cu-overloaded patients. Cells treated with copper ion carriers showed a time-dependent increase of the TCA cycle-associated metabolite dysregulation, suppressing the ETC complex, and inhibiting mitochondrial pyruvate uptake can significantly reduce Cu-induced cell death with no influence on ferroptosis.

### FDX1 is an important regulator of protein lipoylation

The authors identified two mitochondrial proteotoxic stress routes involved in cuproptosis through a genome-wide CRISPR/Cas9 loss-of-function screen. Copper promoted mitochondrial protein lipoylation, a highly conserved post-translational modification targeting lysine, only occurs in four enzymes (dihydrolipoamide S-succinyltransferase, glycine cleavage system H protein, dihydrolipoamide branched chain transacylase E2 (DBT), dihydrolipoamide S-acetyltransferase (DLAT)), and all of these enzymes are involved in regulating carbon entry into the TCA cycle. Cu can bind directly to DLAT and promote the oligomerization of lipoylated DLAT, and lipoyl moiety is essential for copper binding. Furthermore, mitochondrial ferredoxin (FDX1) and lipoyl synthase (LIAS) were reported as crucial effectors of cuproptosis, and knockdown of the FDX1 or LIAS genes caused the accumulation of α-ketoglutarate and pyruvate, which in turn decreased the accumulation of lipoylated DLAT and conferred resistance to cuproptosis. Interestingly, the tight connection between cuproptosis and both FDX1 expression and DLAT protein lipoylation disappeared in the presence of high levels of elesclomol (>40 nM).

**Fig. 3:**
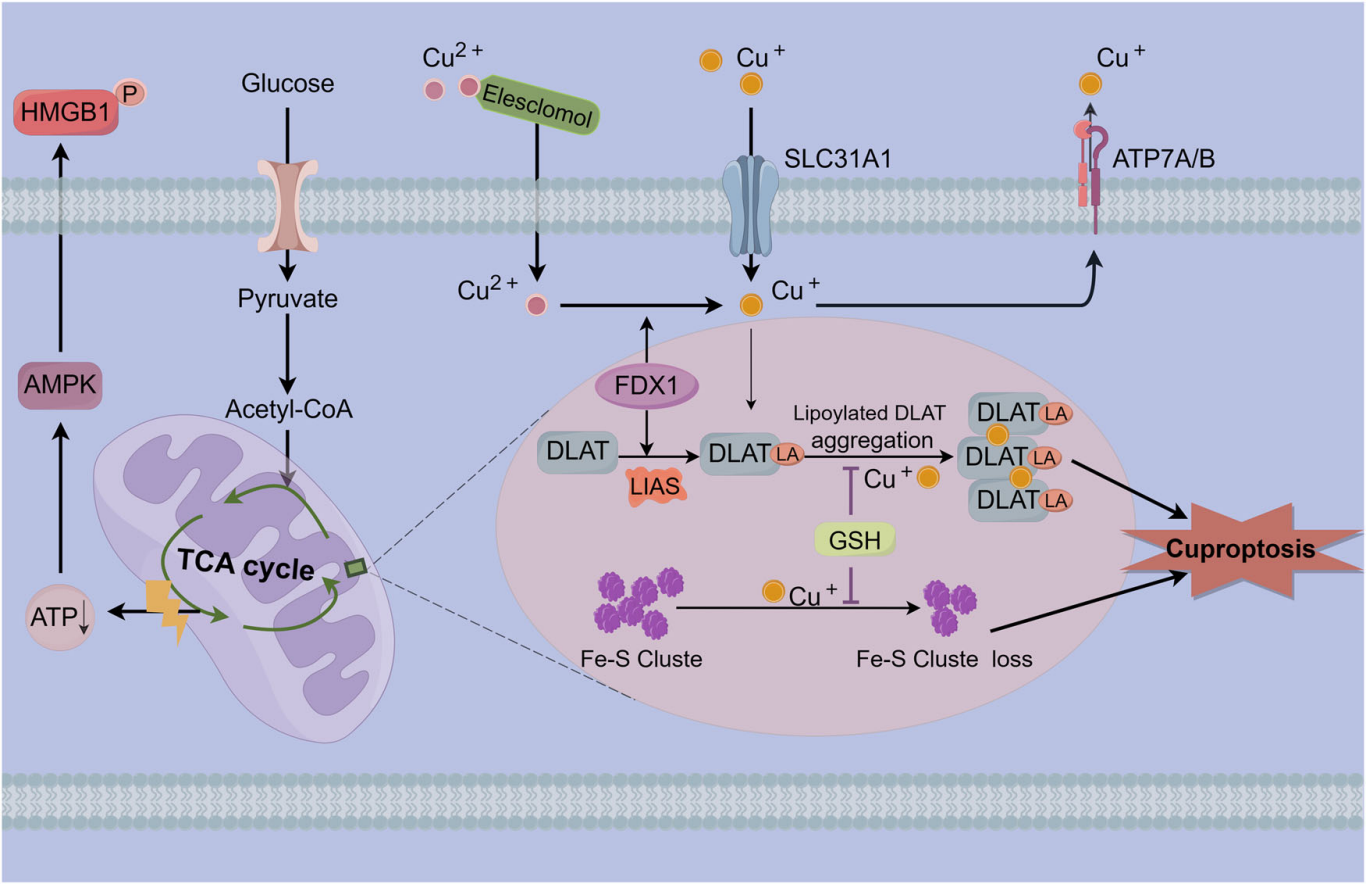
Schematic of cuproptosis mechanism. Extracellular Cu is bound by Cu ionophores like elesclomol and moved into intracellular compartments. Cu then attaches itself to lipoylated mitochondrial TCA cycle enzymes, like DLAT, causing these proteins to aggregate. As the upstream regulator of protein lipoylation, FDX1/LIAS promotes the loss of Fe-S clusters and the aggregation of mitochondrial proteins. These aberrant processes ultimately result in cell demise via proteotoxic stress. Copper chelators such as GSH inhibit cuproptosis.

Notably, besides being associated with aberrant oligomerization of lipoylated proteins, copper toxicity is also closely linked to the destruction of iron-sulfur-containing enzymes. FDX1 is considered the most relevant gene with ES sensitivity, a recent study demonstrated that copper ionophore treatment of cells resulted in the loss of FDX1-dependent Fe-S cluster proteins. Most of the Fe-S proteins will participate in the mitochondrial respiratory ETC and several other biochemical processes as cofactors for enzymes. For this reason, aberrant oligomerization of these copper-bound lipoylated mitochondrial proteins may disrupt the function of Fe-S cluster proteins, inducing proteotoxic stress and ultimately leading to cell death (Fig. 3). Nevertheless, mechanistically, how the aggregation of copper-bound, lipoylated mitochondrial enzymes leads to such nonapoptotic forms of cell death remains to be fully clarified.

## Copper homeostasis and cuproptosis in CNS disease

**Fig. 4:**
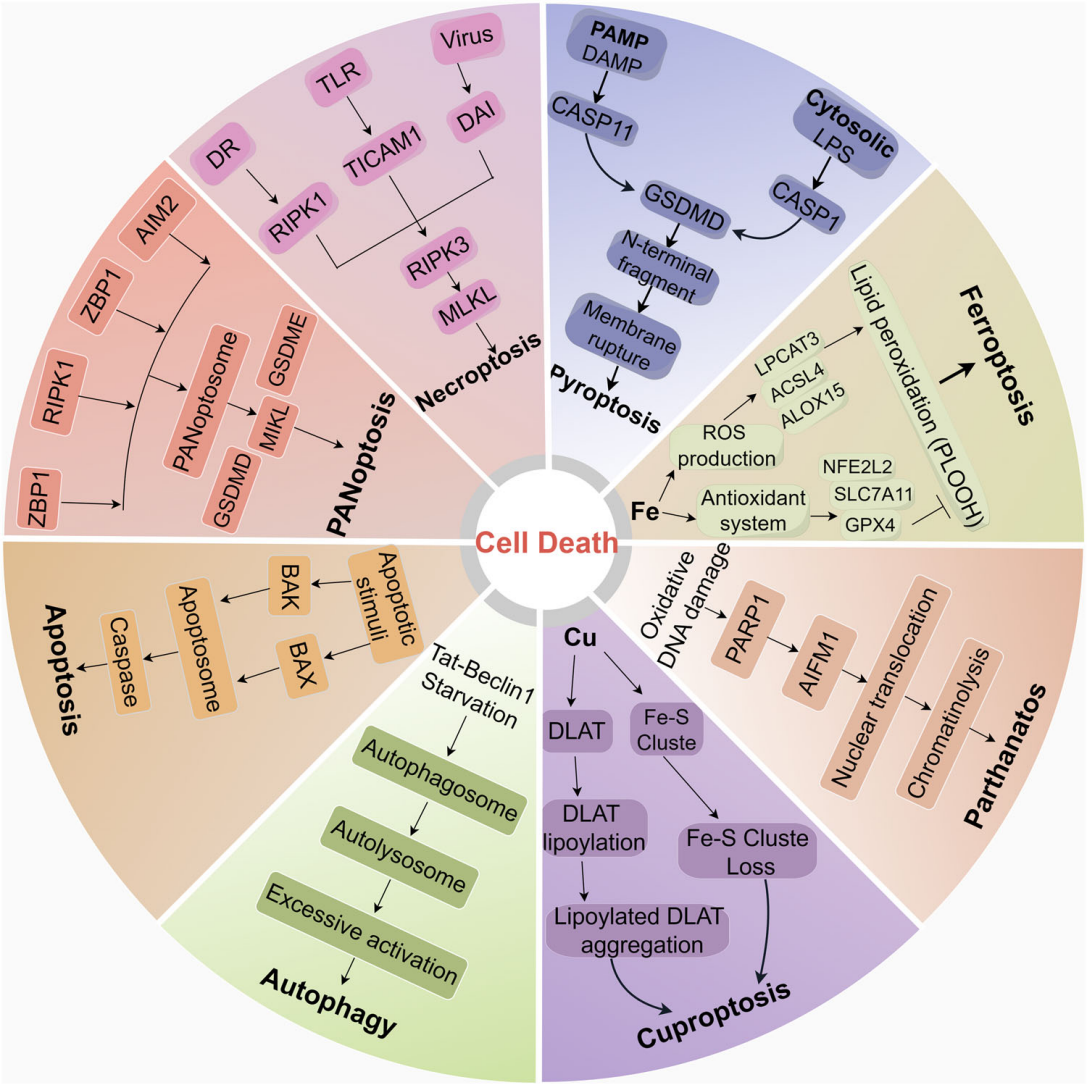
Several common modes of cell death and their major mechanisms.

Cuproptosis is one of the hotspots of current research, and its related molecular mechanisms become increasingly clear due to scholars’ increasing cognizance of the importance of copper homeostasis and copper-induced cell death in disease. Recently, copper-induced cell death has been more studied in cardiovascular cerebrovascular disease and tumor-related disease [48, 49]. Moreover, numerous studies showed that alterations in copper homeostasis are related to the progression of multiple CNS diseases, including gliomas, ALS, AD, Parkinson’s disease (PD), HD, stroke, and so on [10, 50–53]. Chronic copper exposure leads to the accumulation of ROS, which destroys double-stranded DNA, decreases mitochondrial membrane potential, and ultimately causes nerve damage [54]. And cuproptosis is linked closely to nerve damage. The mechanism of cuproptosis is distinct from any previous form of cell death (Fig. 4). Therefore, an in-depth exploration of the regulatory mechanisms of copper-induced cell death in CNS disease may contribute to improved management of CNS disease. In the following, we systematically review the relationship between copper homeostasis imbalance (overload or deficiency) and the copper-induced cell death-associated pathways in CNS disease, hoping to unveil novel promising targets and therapeutic maneuvers for CNS disease.

### Glioma

Copper death and the development and treatment of tumor diseases is one of the current research hotspots. As the most common malignant disease in the CNS, the link between glioma and copper death has attracted the interest of many researchers. Glioma is a primary tumor that originates from neuroglial stem or progenitor cells [55]. It is frequently categorized as WHO grades I–IV based on tumor progression and split into oligodendroglioma, astrocytoma, and ventricular meningioma based on histologic appearance [56]. A higher degree of malignancy is correlated with higher grades. About 80% of brain malignant tumors are gliomas, of which less than 5% survive after five years [57]. Cancer development is intricately connected to the disturbance of copper homeostasis. Cancer cells exhibit a greater demand for copper in comparison to normal tissue cells [48]. Prior research has demonstrated that copper plays a pivotal function in cellular signaling [58]. In addition to promoting endothelial cell proliferation, copper exhibits properties that facilitate angiogenesis. Copper is also essential for the activities of lysyl oxidase, which induces the epithelial-mesenchymal transition (EMT) [59]. Hence, it is unsurprising that copper can contribute to the development of cancer by promoting angiogenesis, cancer cell metastasis, and proliferation [60]. Modulating copper levels in the tumor microenvironment has emerged as a novel strategy for tumor therapy.

Copper chelators decrease the concentration of copper in tumor cells, thus impeding the proliferation and dissemination of cancer cells. The copper chelator tetrathiomolybdate (TTM) can effectively lower copper levels, which stops tumors from growing and spreading [61]. It also decreases tumor angiogenesis [62]. In contrast, copper ionophores can transport extracellular copper to cellular mitochondria and in this way induce cancer cell cuproptosis, which has emerged as a special anticancer strategy, surpassing copper chelators in clinical research. Copper ionophores such as chloroquine and DSF have been clinically trialed as anticancer drugs. Wang et al. have shown that inhibiting the transportation of Cu2+ can reduce the synthesis of ATP in cells [63]. This, in turn, activates the AMPK signaling pathway, decelerates the adipogenesis process, and ultimately halts the proliferation of tumor cells. Copper can hinder the biological functions of cancer cells, triggering PCD by enhancing the generation of ROS [48]. The ability of copper to cause non-apoptotic cell death in glioblastoma stem-like cells (GSC) and their differentiation into endothelial-like cells (GdECs) through the use of the copper ionophore ES was confirmed in addition to its ability to induce apoptosis [64]. The primary mechanism by which ES impacts GSC and GdEC is oxidative stress. DSF, a different copper ionophore, has similarly been demonstrated to induce cytotoxicity via oxidative stress [65]. This non-apoptotic cell death may be closely linked to the recent discovery of cuproptosis. GSC exhibits high-level aerobic respiration, and some drug-resistant tumors also display heightened mitochondrial metabolism [66]. Treatment of certain drug-resistant tumors using 5-fluorouracil or cisplatin revealed that all of these tumor cells exhibited high levels of mitochondrial respiration, which is thought to correlate with tumor drug resistance [67]. Furthermore, it was observed that tumor cells subjected to anti-tumor medications, such as PI, underwent a transition to a condition characterized by heightened mitochondrial metabolism [68]. The mechanism of cuproptosis was mentioned earlier as affecting the TCA cycle, and therefore it is highly dependent on mitochondrial respiration. Combining these specific anticancer medications with copper ionophores can enhance their efficacy in treating cancers that rely on mitochondrial respiration. Ultimately, the induction of cuproptosis by copper ionophores holds greater therapeutic potential for tumor cells with elevated mitochondrial metabolism. Therefore, a potential therapeutic strategy involves determining how to induce tumor cells to transform high mitochondrial metabolism to stimulate their cuproptosis. Surely, the feasibility of treating specific tumors by inducing tumor cell cuproptosis needs to be verified by more clinical studies. Tumor-associated macrophages (TAMs) are attracted to gliomas and facilitate the development of an immunosuppressive milieu. Copper could be involved in the function and recruitment of TAM. Targeting copper to alter the immunological milieu may lessen TAMs’ immunosuppressive effects and increase the efficacy of immunotherapies [69]. Histone Deacetylases (HDACs) are enzymes that remove acetyl groups from histones, affecting chromatin structure and gene expression. Some HDACs require copper to function. By employing copper-targeted tactics to inhibit HDACs, glioma cell proliferation, differentiation, and survival may be suppressed through alterations in gene expression [70]. Although these copper-associated molecular targets above seem to be effective in the treatment of gliomas, we have to admit that these researches lack sufficient clinical persuasiveness.

### Alzheimer’s disease

In addition to CNS tumors, there is a strong link between neurodegenerative diseases and copper. AD is one of the most common progressive neurodegenerative disorders which is characterized pathologically by the accumulation of amyloid plaques and neurofibrillary tangles (NFTs) in the gray matter, essentially blocking neuron communication [71, 72]. The underlying cause is amyloid-beta (Aβ) peptides and Tau protein aggregation due to the abnormal processing of amyloid precursor protein (APP) [73]. Mounting evidence has suggested that dysregulation of copper homeostasis may be relevant to the pathogenesis of AD [74]. Important AD-related components, like as Tau and Aβ, can interact with copper. This theory is supported by the metal hypothesis of AD. Significantly, copper can influence the amyloid cascade by interacting with amyloid APP via a copper-binding domain [75]. Excessive amounts of copper can directly attach to Aβ peptides with a strong attraction, hence promoting the production of Aβ oligomers and leading to heightened neurotoxicity. Moreover, the ratio of free Cu in serum to Aβ deposition was found to be 1:1 in the study reported by Bagheri et al. [76] The binding of Aβ with Cu results in the formation of synaptotoxic dityrosine crosslinking of Aβ dimers. This inhibits the disintegration of dimers and promotes the accumulation of amyloid plaques [77]. In vitro, blocking the binding of Cu to Aβ peptides leads to Aβ degradation, prevents the generation of hydroxyl radicals (•OH), minimizes oxidative damage, and eventually decreases cell death [78].

Microglia are the predominant immunocytes in the CNS. Dysfunction of microglia is believed to be a pivotal element contributing to the cognitive impairments and neurodegenerative processes observed in AD patients [79]. Alternately activated (M2) microglia have anti-inflammatory and restorative properties, in contrast to traditionally activated (M1) microglia, which promote inflammation, in AD [80]. This classification is based on specific cell surface components. The course of AD is largely influenced by the impaired polarization of microglia and the excessive activation of the M1 phenotype, along with the defective M2 phenotype. Previous study found that excessive copper exposure disrupts the equilibrium within microglia, leading to their transformation into the M1 phenotype. This transformation accelerates cognitive decline and the advancement of Aβ [74]. Copper activates the NF-κB signaling pathway in mouse BV-2 cells, which belong to a kind of microglial cell line. This leads to the release of inflammatory mediators such as nitric oxide, tumor necrosis factor, interferon, and interleukin, which in turn stimulates neuroinflammatory processes [81]. Additionally, copper-deficient drinking water disrupted the equilibrium of Aβ amyloid in AD transgenic mice with overexpressed APP. Cu-Aβ complexes inhibited the clearance of neurotoxic Aβ, delayed the expression of lipoprotein receptor-related protein 1, and increased the accumulation of Aβ [82]. Scientists conducted experiments on rodents modeling the disease to verify the therapeutic efficacy of copper-lowering therapies for AD. According to the findings, copper chelators and copper transporter protein inhibitors significantly reduced neuronal cell mortality, ameliorated neurocognitive deficits, and averted Aβ deposition [83, 84]. Copper might induce cuproptosis, disrupt synaptic plasticity, and inhibit CREB/BDNF signaling, causing cognitive impairment in mice [11].

### Amyotrophic lateral sclerosis

ALS, a neurodegenerative disease, shares some similarities and differences with AD. Studies related to copper homeostasis and cuproptosis in ALS cannot be ignored. A fatal neurodegenerative disorder, ALS impacts the CNS. It is distinguished by neuronal degeneration and manifests as dysphagia, respiratory distress, and motor impairments in the extremities [85]. Mitochondrial dysfunction, cytoskeletal defects, altered proteostasis, and impaired RNA metabolism are the four primary pathophysiological processes of ALS [86]. In ALS, difficulties arise in the interaction between CCS and mutant copper-zinc SOD1 [87]. More than 170 mutant SOD1s are associated with ALS, and the pathogenicity of SOD1 mutant proteins results from increased toxicity as opposed to loss of physiological function [88]. As an important copper-binding protein, mutant SOD1 is bound to CCS, resulting in reduced copper delivery to mitochondria, instability of SOD1 accumulation, and limited ROS clearance, ultimately leading to motor neuron toxicity injury [89]. It has been demonstrated that copper deficiency exposes mutant SOD1 to hydrophobic residues, which exacerbates its neurotoxicity; copper supplementation reversed this aberrant alteration. Copper was inadequately bound to more than half of the mutant SOD1 protein in the spinal cord of SOD1G37R mutant mice. Copper-containing compounds that were added resulted in a greater quantity of copper-bound SOD1. The degree of copper deficiency in the SOD1 polymer is thought to be proportional to the clinical severity of ALS [90].

In addition, disruptions in cellular copper homeostasis and compromised cuproenzyme activity may contribute to the exacerbation of SOD1 mutant protein toxicity and the advancement of the disease. Elevated copper concentrations were detected in the cerebrospinal fluid of ALS patients [91]. CCS overexpression accelerated the progression of advanced disease stages in SOD1G93A rodents [92]. COX activity was significantly reduced in mice overexpressing CCS, whereas mice not overexpressing CCS had no change in their COX activity. This indicated that increased delivery of copper to SOD1 and decreased delivery of copper to other cuproenzymes, such as COX, were the results of overexpression of the copper chaperone CCS.

Both copper chelators, such as TTM and D-penicillamine, and copper delivery agents, such as CuII(atsm), have been employed efficiently in several mutant SOD1 animal models [93, 94]. TTM reduces the aberrant clustering of SOD1 mutant proteins together and inhibits their activity. This minimizes the degree of skeletal muscle atrophy and the death of motoneuron cells in SOD1G93A mice [95]. CuII(atsm) has been shown in SOD1G37R and SOD1G93A ALS model mice to enhance motor function and lower mortality [96, 97]. As a copper chelator, D-penicillamine may postpone the onset of ALS model mice’s disease and increase their survival duration [98].

### Stroke

Stroke is another common neurological ailment in addition to tumor and neurodegenerative diseases. Stroke is the second greatest cause of death for adults worldwide, behind ischemic heart disease, and the third leading cause of disability. Stroke affects one in four individuals worldwide [99]. Stroke is a term used to describe neurologic impairment brought on by acute localized CNS injury from vascular sources. Stroke typically encompasses two primary types: ischemic stroke and hemorrhagic stroke, with the former being more prevalent [100]. Prior research has demonstrated that elevated plasma copper levels confer an increased susceptibility to stroke [101]. Additionally, related animal studies have found that chronic copper intake leads to decreased angiogenesis and aggravated ischemic injury in mice [102]. Based on the 2013–2018 U.S. National Nutrition Examination Survey, a case-control study found that people who ate more copper had a lower risk of stroke [103]. In a different controlled study, Yang and his colleagues [104] found that plasma copper was strongly linked to a higher risk of ischemic stroke but not significantly linked to a higher risk of hemorrhagic stroke. Another meta-analysis revealed elevated blood copper levels in the ischemic stroke group as compared to the control group, indicating that a high serum copper level is a potential risk factor for ischemic stroke [105]. These seemingly contradictory findings may be explained by the pro- and anti-oxidant characteristics of copper. Moderate levels of copper are beneficial to an organism’s normal physiological metabolism; nevertheless, excessive amounts of copper can be poisonous to organisms. These studies lacked uniformity regarding sample demographics, stroke subtypes, and copper content. Additionally, there were variations in the important confounders and biases.

Copper is a crucial co-factor in various physiological metabolic and redox reactions, playing an indispensable role in the functioning of cuproenzymes including SOD1 and CCO [106]. SOD1 can lower the amount of ROS in the cerebrum, which helps neural stem cells survive, protects the rat brain from cerebral ischemic injury, and reduces the risk of ischemic stroke [107]. The functional profile of SOD1 is opposite when it is misfolded. Consequently, it is reasonable to hypothesize that suppressing these mutated enzymes could prevent or attenuate cuproptosis, potentially enhancing the prognosis of stroke patients. Excessive copper may also worsen the damage caused by ischemic stroke. Jiang et al. found that copper stopped endothelial progenitor cells from dividing. It blocked angiogenesis by raising the amount of thrombospondin-1, which made ischemic strokes in mice worse [108].

In addition, cuproptosis-related mitochondrial dysfunction is also strongly associated with stroke. Hence, in addition to regulating copper homeostasis, improving mitochondrial dysfunction is also crucial in treating stroke. Previous studies have proposed that mitochondrial dysfunction is associated with reduced ATP synthesis in ischemic stroke, ATP deficiency leads to neuronal cell death. Cuproptosis is considered a critical mechanism of nerve cell death due to its reliance on mitochondrial respiration; however, there is insufficient evidence to support this claim [109]. Huo et al. [110] propose that cuproptosis is the result of non-specific interactions between copper and lipoylated proteins in mitochondria. This interaction also diminishes the normal expression of proteins in the mitochondrial oxidative respiratory chain, which in turn results in reduced levels of ATP and oxygen. Given the high demand for ATP and oxygen in brain tissue, it is necessary to investigate whether cuproptosis may play a significant role in stroke. At present, there is no definitive clinical drug that can treat stroke by modulating cuproptosis. Nevertheless, these conjectures and findings offer some direction for the development of future pharmaceuticals.

### Huntington’s disease

HD is an autosomal dominant progressive neurodegenerative disease with clinical manifestations of cognitive deficits, motor difficulties, and dystonia [111]. Mutant huntingtin proteins (mHTT) in HD, caused by aberrantly amplified CAG repeat sequences resulting in long-chain N-terminus polyglutamine production. Neurodegeneration and oxidative stress are induced by the cytotoxicity of N-terminus mHTT fragments [111].

Copper has been implicated in the pathogenic mechanism of HD. Abnormally high concentrations of copper were found in the striatum of HD patients and the brains of HD model mice [112]. Copper can interact with metal binding sites on mHTT monomers and promote mutant Huntington protein aggregation in mice [113]. Using a copper chelator (bathocuproine disulfonate) can effectively modulate the occurrence of early events of HTT misfolding and reduce neurotoxicity in Drosophila models of HD [114]. Increasing copper concentration promotes HTT aggregation and facilitates HD disease progression. Additionally, TTM and Clioquinol can significantly delay neuropathological processes in HD mice [115, 116].

## Potential therapeutic strategies for CNS disease

Restoring copper homeostasis to reduce its caused neurotoxicity and cell death has emerged as a potential treatment strategy for CNS illnesses due to the impact of copper homeostasis imbalance and cuproptosis in these conditions. Drugs targeting the regulatory mechanisms involved in cuproptosis and interrupting its occurrence are expected to be new drugs for refractory CNS diseases. Currently, there is a growing study on the use of copper chelators to reduce the harmful effects of copper overload and manipulate the levels of copper within and outside cells. Moreover, Current approaches to addressing the problems associated with cuproptosis mostly rely on copper chelators and copper ionophores. Furthermore, nanomaterials are also being used to provide more choices for the therapies of CNS disease. In the following content, we discuss therapeutic strategies that target copper homeostasis and cuproptosis in CNS disease in more detail.

### Copper chelators

Significant promise was demonstrated by copper chelators in the management of CNS disease (Table 2). Maintaining intracellular copper homeostasis via copper chelators is particularly important in gliomas, AD, and HD. Copper chelators, such as D-penicillamine, TTM, Triethylenetetramine (TETA), Trientine, Ethylenediaminetetraacetic Acid, and Batocuproin, are frequently employed in clinical and experimental settings. D-penicillamine is one of the most commonly used copper chelators for treating Wilson’s disease, effectively reducing free copper levels in the blood and tissues, and was the first FDA-approved copper chelator drug for treating Wilson’s disease [117]. D-Penicillamine chelates copper via the formation of stable cyclic complexes with copper and thereby maintains copper homeostasis in the body. In addition, it destabilizes copper ions, which alters the expression of CTR1 and MT3 in the choroid plexus, consequently reducing intracellular copper levels [118]. Moreover, D-penicillamine may have immunomodulatory effects and be useful in treating some autoimmune conditions. Adverse events, such as myelosuppression, nephrotoxicity, and hypersensitivity reactions (rash, fever), are typical with D-penicillamine. In individuals with Wilson’s disease, it may exacerbate neurological symptoms, particularly in the early stages of treatment. Furthermore, excessive D-penicillamine exposure in pregnant women can result in aberrant fetal development. 8-Hydroxyquinoline is a lipophilic metal chelator with a significant inhibitory effect on Cu-induced abnormal Aβ aggregation and alleviates disease progression in AD [119]. Trientine is less likely to have adverse effects than D-penicillamine, and it is also less likely to exacerbate neurological symptoms in Wilson’s disease patients. Additionally, it can be effectively utilized in individuals who are intolerant to D-penicillamine. Nonetheless, it remains challenging to prevent its gastrointestinal adverse effects [120]. TTM, a small hydrophilic compound, functions by impeding angiogenesis in the tumor blood supply region through the reduction of copper concentrations. TTM also exhibits an extraordinary therapeutic impact when applied to the treatment of Wilson’s disease. TTM has a lower risk of early neurological impairment than D-penicillamine and is also effective at lowering free copper levels. In addition to this, TTM has the dual action of both chelating copper and blocking its absorption. Nevertheless, TTM remains in the experimental phase, has not received approval from pertinent regulatory agencies, and there is a dearth of long-term safety evidence. Compared to D-penicillamine or Trientine, it is also more difficult to obtain [121]. In addition to being a metal chelator with a notable affinity for Cu, TETA is employed as a second-line medicine for Wilsom’s disease [117]. Also, it has been revealed that TETA inhibits angiogenesis and ameliorates the progression of gliomas. Although the copper chelator clioquinol is difficult to penetrate the blood-brain barrier due to its hydrophobicity, it has been demonstrated to reverse amyloid deposition in APP-transgenic mice. TETA is less nephrotoxic than the copper chelators mentioned above, although its useful effects have only been shown in limited clinical trials.

**Table 2:** Application of Cu chelators in cuproptosis-related CNS diseases.

Cu chelator	Type of model/subject	Mechanism	Ref
TTM	Aβ/PS1 transgenic mice	Promotion of non-amyloidogenic protein production of AβPP	[147]
TTM	BV-2 microglia	Reducing Inflammation by Inhibiting the TRAF6/AKT/NF-κB signaling pathway	[148]
TTM	Transgenic mice expressing human mutant SOD1	Reduces copper ion levels and inhibits lipid peroxidation	[93]
D-penicillamine	AD patients	Reduces oxidative stress damage	[149]
D-penicillamine	AD model In vitro	Solubilization of Cu-Aβ with assistance of nanoparticles	[150]
D-penicillamine	Stroke	Improving myocardial function and reducing ischemic events	[151]
D-penicillamine	Glioblastoma multiforme	Inhibits tumor cell proliferation through the TGF-β/Smad signaling pathway	[152]
TETA	Transgenic mouse model of AD	Reduces BACE1 activity and mitigates amyloidosis	[153]
Clioquinol	U87 glioblastoma cells	Induction of apoptosis and autophagy in glioblastoma	[154]
Clioquinol	transgenic Huntington’s mice (R6/2)	Inhibition of HTT aggregation and reduction of striatal atrophy	[115]
Clioquinol	Twenty AD patients	Reduces levels of CSF-Tau protein	[155]
8-hydroxyquinoline	Glioma	Activates transcription factors in glioma cells and inhibits cell proliferation	[156]
8-hydroxyquinoline	AD	Inhibits cholinesterase and reduces Aβ levels	[152]

Undoubtedly, metal chelators offer advantages in enhancing the progression of disease, but their drawbacks should not be overlooked. Studies have demonstrated that metal chelators facilitate the transportation of heavy metals from peripheral tissues to the brain, hence enhancing the generation of neurotoxic substances [122]. Natural detoxifiers are more cost-effective and readily available than the traditional metal chelators mentioned above, typically resulting in fewer adverse effects. Curcumin possesses the ability to chelate copper and other metal ions robustly, and it is neuroprotective in the treatment of numerous CNS diseases, including AD and PD [123]. In addition, Huang et al. [124] discovered a natural compound from Streptomyces sp. CB00271 can bind to copper, which they call chalkophomycin (CHM, 1). Chalkophomycin is thought to have potential therapeutic value as an anti-neurodegenerative disease and anti-tumor. Generally speaking, copper chelation therapy remains in the experimental phase and is far from being clinically applicable to patients with CNS disease.

### Copper ionophores

Unlike copper chelators, copper ionophores like ES and DSF facilitate the transport of copper into cells to regulate intracellular copper levels. ES, a crucial component of the cuproptosis mechanism, specifically acts on mitochondrial respiration. Its therapeutic potential is due to its ability to potentially induce cuproptosis in cancer cells, such as gliomas [125]. DSF inhibits NF-κB activity, specifically targets glioblastoma stem cells produced by hypoxia, and blocks their proliferation and invasion [126]. Traditional copper ionophores have some non-negligible shortcomings. First of all, copper ionophores dominate the transport process, making it difficult to achieve targeted and accurate regulation. Once overloaded with transported copper it may lead to oxidative stress damage by triggering the Fenton chemical reaction. Furthermore, the unselective and thoughtless application of copper ionophores to address deficient copper levels in particular tissues or organs can result in the incorrect buildup of copper [127]. Therefore, it is a challenge to regulate the transportation of copper efficiently and accurately. Su et al. [128] provided a novel modular platform designed to deliver metal ions to specified tissues. An ionophore functionalized with N-acetylgalactosamine, called Gal-Cu, is intended to operate as a “Trojan Horse” that carries copper. This concept provided new thoughts on the application of metal ions in CNS diseases. Presently, the utilization of copper-based nanomaterials for treating disorders linked to copper homeostasis has emerged as a prominent area of research.

### Copper nano-complexes

**Fig. 5:**
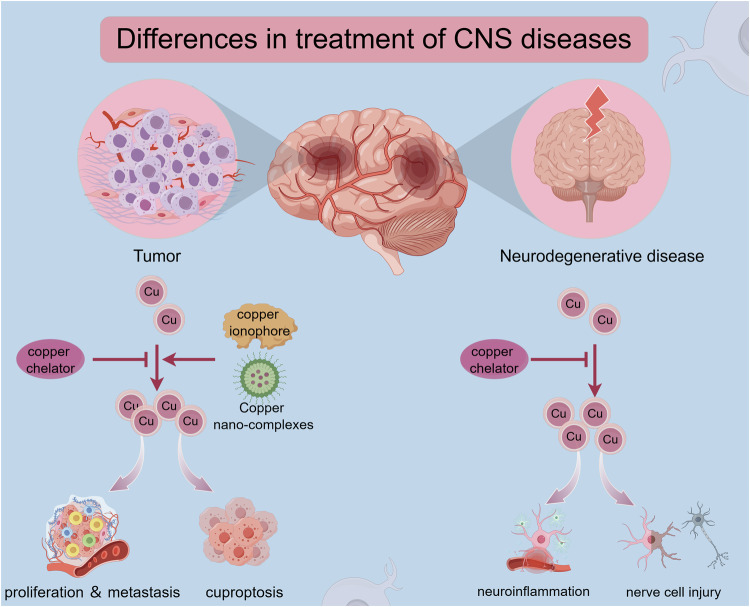
Therapeutic focus of different diseases. Copper overload promotes the progression of neurodegenerative diseases, with excess copper accumulating in diseased areas, leading to nerve cell death, neuroinflammation, and the development of oxidative stress. The therapeutic strategy focuses on the removal of copper accumulation at the lesion site by copper chelators to reduce neurotoxicity. However, it is not quite the same with tumors. On the one hand, copper promotes the proliferation and metastasis of tumor cells. The progression of tumor cells can be inhibited by copper chelators. On the other hand, large amounts of copper can induce cuproptosis in tumor cells. Tumor growth can be inhibited by copper ionophores and Cu-based nanomaterials.

Different diseases have different pathogenic mechanisms and their treatment varies (Fig. 5). As previously stated, modulating copper homeostasis by applying copper chelators or copper ionophores is viable. However, it doesn’t seem that taking medicine orally produces positive effects [129]. Currently, the application of traditional copper ionophores and copper chelators in treating CNS disease remains restricted. The challenges in treating these diseases may be attributed to the inadequate absorption of oral medicines and the inherent constraints of the blood-brain barrier. In addition, the use of copper ions in biomedical applications is severely limited by their metallic toxicity. The advent of nano complexes encapsulating therapeutic drugs seems to have improved the current situation. Copper-based nanomaterials are not transported in an ionic state when used in the body, effectively mitigating the toxic impairment caused by copper ions to normal cells. Even though nanoparticles have not yet been implemented in clinical applications, their utilization has broadened the possibility of treating refractory CNS diseases, especially cancer **(**Table 3).

**Table 3:** Application of copper nano-complexes in CNS diseases.

Copper nano-complex	Type of disease/subject	Mechanism	Ref
Au@MSN-Cu/PEG/DSF	Tumor cell	Biodegradation induced by photothermal induction of material carrying Cu and DSF-inducing tumor cell cuproptosis	[140]
L-/D-Pen@Se NPs	Alzheimer’s disease	Inhibition of copper induced Aβ deposition	[133]
MXenzyme	Alzheimer’s disease	Chelates copper and scavenges ROS to reduce Aβ deposition	[157]
HSA-BFP@CDs	Alzheimer’s disease	Detects and inhibits Aβ aggregation, mitigates oxidative stress	[158]
PEG-PAsp(DET)	Ischemic stroke	Targeted repair of vascular injury after ischemic stroke	[159]
nano IVA	Huntington’s disease	Activates cellular autophagy and reduces neuronal damage	[149]
CuO-NPs	C6 glioma cells	Induction of cytotoxic injury in C6 glioma cells by copper accumulation	[130]
Au@Cu2-xSe NPs	Glioblastoma	Inhibition of protective autophagy and DNA repair in glioma cells enhances the efficacy of radiotherapy	[160]
CuHARS	CRL2303 Glioma	Increased inflammatory stressors induce glioma cell necrosis	[161]
AngMNPs@ (Dp44mT/Reg) NPs	glioblastoma multiforme	Targeted increase of copper concentration in tumor cells induce apoptosis	[162]
^64^Cu-AuNCs	Pontine gliomas	Quantifying the efficiency of targeted drug delivery	[132]

Nanomaterials have the potential to revolutionize CNS disease management drug delivery by effectively crossing the BBB and improving drug delivery efficiency. When utilized in the synthesis of copper complexes, nanomaterials can more efficiently regulate copper levels. Thus far, researchers have developed a variety of integrated copper-based nanocomplexes with distinct nanostructures and applied them to cancer treatment. These applications primarily encompass photodynamic therapy, radiotherapy (RT), chemodynamic therapy (CDT), sonodynamic therapy (SDT), photothermal therapy (PTT), and copper-induced cell death. The proliferation of tumor cells is intricate and unpredictable, making it impractical for a solitary therapy method to adequately address the increasing demand for treatment. Hence, the development and utilization of multimodal combination therapy are promising in tumor treatment. Applying copper oxide nanoparticles (CuO-NPs) to C6 glioma cells induces glioma cytotoxicity injury through concentration and temperature-dependent copper accumulation [130]. Besides directly regulating Cu levels, some nanomaterials can be used to detect micromolar changes in Cu concentration. As a contrast agent for PET imaging, DOPA modified by DOTA can chelate copper and assist in detecting micro changes in copper concentration, which provides for treating neurodegenerative disease [131]. On this basis, A was designed to quantify the efficiency of drug delivery for the treatment of pontine gliomas [132]. The cognitive function of AD model mice was significantly enhanced by chiral penicillamine-encapsulated nanoparticles (L-/D-Pen@Se NPs), which Sun et al. [133] demonstrated could inhibit copper-induced Aβ deposition selectively.

These nanomaterials usually need to be stimulated to start working. Endogenous stimuli such as enzymes, ROS, and pH may be involved in controlling drug delivery to the tumor site to improve efficacy [48, 134–136]. Nevertheless, these endogenous biomarkers are present in both the tumor microenvironment and the normal microenvironment with low levels and suffer from poor specificity and low release efficiency. External energies such as ultrasound, X-rays, and light may offer better strategies for precise drug delivery [137–139] **(**Table 4**)**. Zhou and colleagues [140] developed a photothermally triggered nanoplatform, Au@MSN-Cu/PEG/DSF, with the capability of inducing cuproptosis in tumor cells and impeding tumor growth (Inhibition rate reached 80.1%). In addition to photosensitizers, sonosensitizers have also been employed extensively in the construction of copper nano-complexes. SDT shows great potential as a non-invasive treatment for cancer, as it may effectively penetrate deep into tissues and provide precise targeting. SDT utilizes ultrasound to activate sonosensitizer generating massive amounts of ROS inducing oxidative stress, ultimately causing cancer cell death. Ultrasound-triggered SDT has greater tissue penetration compared to light-mediated therapy, making it a more effective non-invasive treatment option for deep-seated tumors like glioblastoma. Besides participating in constructing copper nano-complexes, Cu-based nanoparticles can also be used as a sonosensitizer [129]. Zhu and his colleagues [141] constructed carrier-free nanoparticles (Ce6@Cu NPs) utilizing the coordination assembly of Cu^2+^ with sonosensitizer chlorin e6. Ce6@Cu NPs accumulate effectively within U87MG cells. Following exposure to ultrasound, the NPs initiated oxidative stress, depleted endogenous GSH, and caused the aggregation of DLAT, ultimately leading to cuproptosis in tumor cells.

**Table 4:** Stimuli for the induction or enhancement of copper-based nanomaterials.

Stimulus	Nanocomplexes	Therapy methods	Tumor cells	Ref
Light	Au@MSN-Cu/PEG/DSF	PTT/Cuproptosis	4Tl	[126]
	T-HCN@CuMS	PTD	143B	[131]
	CuSiO_3_@Au-Pd	PTT/CDT	MCF-7	[134]
Ultrasound	Ce6@Cu NPs	SDT/Cuproptosis	U87MG	[136]
	Lip@Cu_3_N/PFC-O_2_	SDT/Cuproptosis	—	[137]
X-ray	RCL@Pd@CuZ	RT	MC38	[135]
ROS	CSTD-Cu(II)@DSF	CDT/ Cuproptosis	MCF-7	[48]
	NP@ESCu	Cuproptosis	BIU-87	[48]
pH	TP-M-Cu-MOF/siATP7a	CDT/ Cuproptosis	SCLC	[161]
ROS/pH	CSTD-Cu(II)@DSF	Chemotherapy/ Cuproptosis	MCF-7	[162]
GSH	CuET-NPs	Chemotherapy	A549	[132]

Besides improving treatment efficiency by combining therapies such as PTT, RT, and STD, how to enhance the efficiency of crossing the BBB and accessing brain lesions is also a prominent focus of current research. Chen et al. utilized neutrophils to envelop the combination of thermosensitive liposomal and Cu-Fe bimetal (Lip@Fe-Cu-MOFs). This research has demonstrated its efficacy in treating breast cancer in mice and its ability to target specifically and induce cuproptosis in tumor cells [142]. This approach could be utilized in the domain of brain tumors to mitigate the impact of the BBB on the permeability of medication. In summary, current researches about copper nano-complexes focus more on targeting and specificity.

Copper nano-complexes have demonstrated significant potential in the treatment of tumors. Nevertheless, clinical translation continues to pose substantial obstacles. (1) Biocompatibility & Toxicity: Prolonged exposure to high concentrations of copper ions can cause non-negligible metal toxicity to the human body, particularly to the liver, kidneys, and CNS. Further research is required to reduce copper ions’ concentration without compromising therapeutic efficacy. (2). Metabolism & excretion: Retention of copper-based nanomaterials in the body can have serious side effects, and it is critical to ensure that these nanomaterials are safely and easily metabolized and excreted by the body. (3) Drug resistance: Tumor cells might acquire resistance to these copper-based nanomaterials, and the resolution of tumor drug resistance is a difficult issue that necessitates extensive research.

## Conclusion and future perspectives

Copper is involved in both healthy and diseased cellular physiology. On the one hand, as a biologically essential trace element, copper is an important cofactor for various enzymes; on the other hand, copper overload can cause oxidative stress damage and even cell death. In recent years, copper homeostasis and copper-induced related cell death in CNS disease have received much focus. The current study has revealed that cuproptosis is a completely new form of cell death dependent on copper, and is mediated by the mitochondrial enzyme lipoylation. This finding provided novel ideas about the relationship between copper-induced cell death and mitochondrial metabolism, as well as new directions for therapeutic strategies for several disorders, including CNS disease. However, in contrast to apoptosis, necrosis, and ferroptosis, research on cuproptosis is comparatively scarce. Further studies are needed to clarify whether cuproptosis plays a linchpin role in the pathogenesis of CNS disorders and whether cuproptosis can be used as an entrance point for the treatment of these refractory CNS disorders.

### Reporting summary

Further information on research design is available in the Nature Research Reporting Summary linked to this article.

## Supplementary information

Reporting Summary
